# MicroRNAs Secreted by the Embryo in Spent Culture Medium Can Regulate mRNAs Involved in Endometrial Receptivity, Embryo Attachment, and Invasion

**DOI:** 10.3390/ijms26188879

**Published:** 2025-09-12

**Authors:** Angela Caponnetto, Carmen Ferrara, Anna Fazzio, Luca Carli, Cristina Barbagallo, Michele Stella, Davide Barbagallo, Marco Ragusa, Michael Feichtinger, Cinzia Di Pietro, Rosalia Battaglia

**Affiliations:** 1 Department of Biomedical and Biotechnological Sciences, Section of Biology and Genetics “G. Sichel”, University of Catania, 95123 Catania, Italy; 2 Wunschbaby Institut Feichtinger, 1130 Vienna, Austria; 3 Department of Medicine and Surgery, University of Enna “Kore”, 94100 Enna, Italy

**Keywords:** embryo culture medium, miRNA, endometrium, cross-talk embryo maternal tissues, implantation

## Abstract

MicroRNAs, secreted by the embryo in blastocoel fluid (BF) and embryo spent culture medium (SCM), regulate important cellular pathways controlling the stemness of inner cell mass, trophectoderm differentiation, and the dialogue between blastocyst and maternal tissues. In recent years, their role as non-invasive biomarkers of embryo quality has been deeply investigated. We compared the expression profiles of 96 microRNAs between BF and SCM from the same embryos, highlighting the differences between these two compartments. We found 10 and 6 microRNAs specifically expressed in BF and in SCM, respectively; 22 microRNAs significantly up-regulated in BF; and 2 significantly up-regulated in SCM. To investigate the role of SCM microRNAs in implantation, we focused on the microRNAs specifically expressed/up-regulated in SCM and absent in blank medium. We deepened our understanding of SCM microRNA’s biological role by building a network of miRNA–mRNA interaction within the signalling pathways crucial in embryo implantation success. We demonstrated that BF and SCM contain different sets of microRNAs playing different and unique roles in embryo implantation and development. Finally, we suggest that there is not a single “ideal” technique to identify the most competent embryo, but an integrated approach is needed to obtain informative results on the health of the embryo.

## 1. Introduction

Identifying the most developmentally competent embryo for transfer into the uterus remains one of the most critical and challenging aspects of Assisted Reproductive Technologies (ARTs). Despite significant advances in in vitro fertilization (IVF) protocols, the success rates of ARTs still depend heavily on the precise identification of embryos with the highest potential to result in a successful pregnancy and live birth [1].

Currently, the primary non-invasive method for identifying viable embryos relies on blastocyst morphological evaluation [2]. However, embryos with good morphology can still harbour aneuploidies or epigenetic abnormalities that compromise implantation or early embryonic development [3].

To improve pregnancy outcomes and reduce the risk of monogenic diseases or aneuploidies in offspring, different pre-implantation genetic testing (PGT), such as PGT-A (aneuploidy), PGT-M (monogenic), and PGT-SR (structural rearrangements) have been developed [4]. However, the trophectoderm biopsy, required for these methods, is an invasive procedure that raises concerns due to its potential to impair embryo viability and implantation. Furthermore, the limited sample size taken from any embryo increases the risk of false-negative or false-positive outcomes [5]. To address these concerns, researchers have increasingly focused on the development of non-invasive genetic testing approaches involving the analysis of cell-free DNA (cfDNA) released by the embryo into both the blastocoel fluid (BF) and the spent culture medium (SCM) [6,7]. The detection of cfDNA offers the potential to assess the genetic status of the embryo without physically breaching its structural integrity [8]. The analysis of SCM is particularly advantageous due to its accessibility and the feasibility of routine sampling in IVF laboratories. While BF aspiration is a minimally invasive method, it allows better access to cfDNA from the inner cell mass (ICM) and is less prone to contamination from cumulus cells, sperm, or culture medium [9]. Even though non-invasive technology avoids the risks linked to biopsy-related damage and reduces the chances of sampling bias, several technical and biological challenges must still be addressed, including the optimization of DNA amplification techniques, the minimization of external contamination, and the establishment of robust criteria for interpreting cfDNA results.

Human embryos, through paracrine and autocrine secretion, release several molecules into the extracellular environment (BF and SCM), including microRNAs (miRNAs). These small non-coding RNAs have emerged as promising candidates due to their regulatory functions in gene expression and their stability in extracellular fluids. The molecules secreted in SCM are part of blastocyst–endometrium interaction, which is necessary for implantation success. This interaction promotes the establishment of phase synchrony, ultimately aiming to create a hospitable environment for competent embryos at the implantation site, which is a prerequisite for successful implantation [10].

MiRNAs possess many critical regulatory functions in a wide range of biological processes, such as cell proliferation, differentiation, survival and apoptosis, and stress responses. A single miRNA has the potential to modulate the expression and functions of several downstream target genes. In addition, the existence of feedback regulation mechanisms between a miRNA, its targets, and their products allows for amplification or inhibition of specific biological and molecular signals [11]. MiRNAs are known regulators of decidualization, endometrial receptivity, and embryo implantation, crucial processes for reproductive success. Their dysregulation could be linked to implantation failure and early pregnancy loss [12,13,14].

Different studies have investigated the correlation between the expression profiles of specific miRNAs and embryonic quality, chromosomal status, and implantation potential [15].

Several studies have demonstrated that the collection and analysis of SCM for miRNA profiling represents a fully non-invasive strategy that avoids direct manipulation of the embryo [16]. However, SCM can contain several miRNAs that are not secreted by the embryo and, thus, represent an important source of contaminants that could have potential biological implications for embryo development [17].

Based on current knowledge, to address the major challenge faced by embryologists of identifying the most viable embryos, different factors must be considered: the embryo morphology; blastocyst formation time; copy number variation; and genetic, epigenetic, and transcriptomic mechanisms. In this context, multi-omics approaches could provide a multidimensional reference for more accurate embryo identification [18]. By adding the transcriptome-based dimension to the genomic level results of PGT-A, it should be possible to provide new insights into the clinical applications of PGT [19].

Building on our previous study demonstrating the regulatory role of miRNAs in BF [20], the present study focuses on identifying mRNAs and the associated signalling pathways regulated by miRNAs specifically secreted into SCM, with the aim of further elucidating their contribution to implantation success.

## 2. Results

### 2.1. MiRNA Expression in Blastocoel Fluid and in Spent Culture Medium

**Figure 1:**
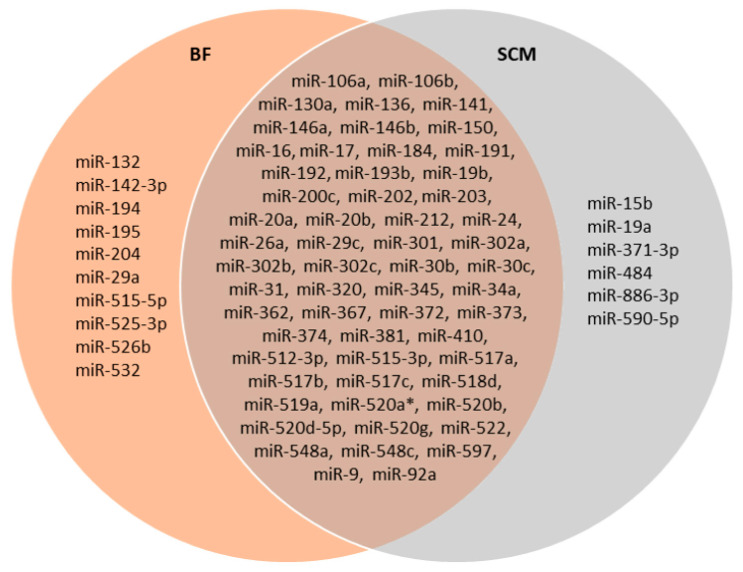
MiRNA detection in BF and SCM. The Venn diagram illustrates the distribution of miRNAs detected in the BF samples in the orange circle on the left, and in the SCM samples in the blue circle on the right. MiRNAs detected in both BF and SCM are displayed in the overlapping central region.

In the comparison between BF and SCM, we found ten and six miRNAs specifically expressed in BF and SCM, respectively (Figure 1). Moreover, fifty-eight miRNAs were detected in both BF and SCM samples (Figure 1). The remaining miRNAs were excluded from the analysis because their Ct values did not match the selection criteria described in the Materials and Methods section.

**Figure 2:**
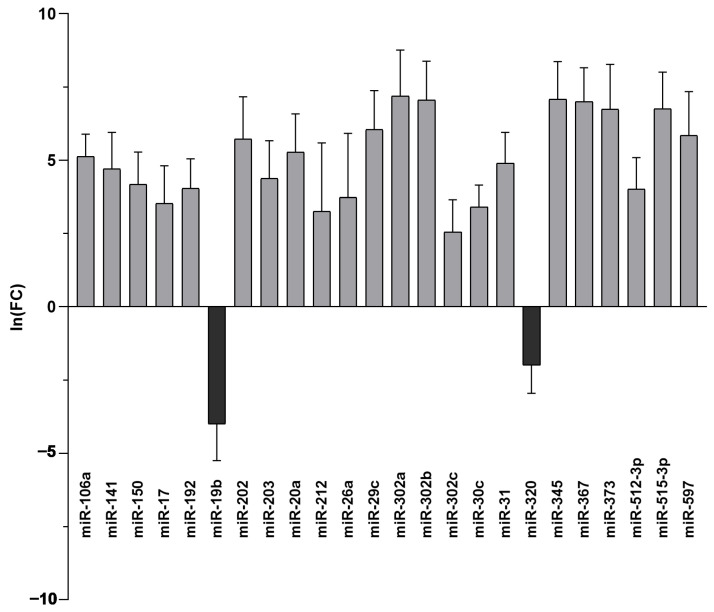
Differentially expressed miRNAs in BF vs. SCM. Twenty-two miRNAs are up-regulated in BF (light grey-coloured bars) and two miRNAs are down-regulated in BF and consequently up-regulated in SCM (dark grey-coloured bars). Ln (FC) values of significantly deregulated miRNAs in BF vs. SCM are reported on the y-axis.

**Figure 3:**
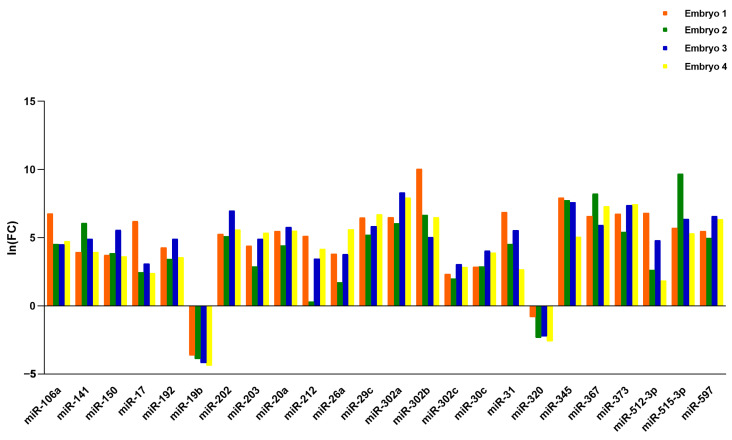
Differentially expressed miRNAs in single embryos. Ln (FC) values of deregulated miRNAs in the single embryos are reported on the y-axis.

Among the fifty-eight miRNAs detected in both BF and SCM samples, we found twenty-four miRNAs to be significantly dysregulated. Specifically, twenty-two miRNAs were up-regulated in BF compared to SCM, while two miRNAs (miR-19b and miR-320) were up-regulated in SCM compared with BF (Table 1 and Figure 2). Notwithstanding individual variability, this miRNA expression trend was confirmed when we compared BF and SCM from the same embryo (Figure 3).

### 2.2. MiRNA Expression in Spent Culture Medium and in Blank Medium

**Figure 4:**
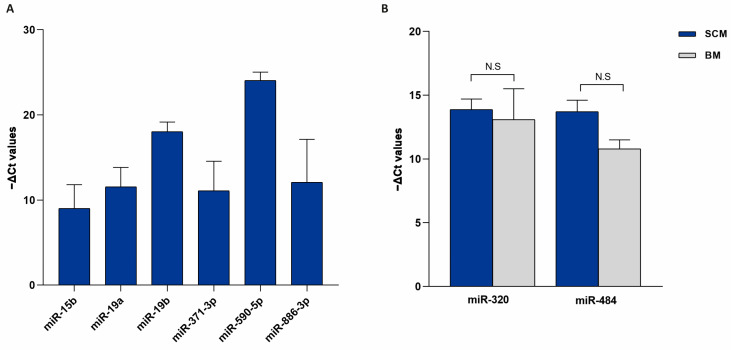
MiRNAs expressed in spent culture medium and in blank medium. (**A**) MiRNAs expressed in SCM and not detected in BM; (**B**) MiRNAs expressed both in SCM and in BM. −ΔCt values are reported on y-axis. N.S = not significant.

In order to evaluate the contribution of possible miRNAs present in blank medium (BM) that could invalidate the results, the expression analysis of specific and/or differentially expressed miRNAs in SCM was performed by comparing SCM samples with BM samples. Mir-15b, miR-19a, miR19-b, miR-371-3p, miR-590-3p, and miR-886-3p were not detected in BM; therefore, their expression levels in SCM were not influenced by BM or by background contamination (Figure 4A). MiR-484 was found to be up-regulated in SCM but did not reach statistical significance (ln (FC) = 2.01 and *p*-value = 0.11), while miR-320 did not vary (Figure 4B). For this reason, these last two miRNAs, representing possible contaminants, were not considered for further analysis.

### 2.3. Network miRNA–mRNA Interaction and Signalling Pathway Analyses

**Figure 5:**
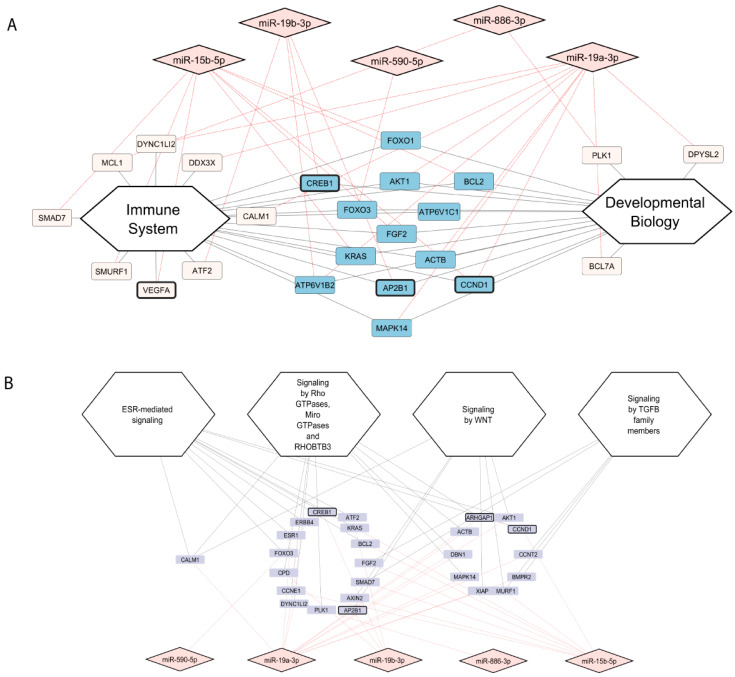
MiRNA–mRNA pathway regulatory networks. (**A**) Network representation of mRNAs associated with the Immune System and Developmental Biology pathways and the miRNAs that regulate them. The nodes coloured in blue correspond to the mRNAs shared between both pathways. Nodes with thick black borders are those found to be deregulated during the window of implantation. (**B**) Network showing mRNAs and their regulating miRNAs involved in the ESR-mediated signalling pathway; Signalling by Rho GTPases, Miro GTPases, and RHOBTB3 pathway; Signalling by WNT pathway; and Signalling by TGFB family members pathway. The mRNAs deregulated in the window of implantation are indicated by nodes with thick black borders.

The targets of the six miRNAs reported in Figure 4A were used to perform the miRNA–mRNA interaction analysis (Figure 5). Among the one hundred and four retrieved mRNA targets, sixty are expressed in the endometrium. Of these, twenty-one mRNAs were found to be involved in the Immune System pathway and sixteen in the Developmental Biology pathway (Figure 5A); ten in ESR-mediated signalling pathway; nine in the Signalling by Rho GTPases, Miro GTPases, and RHOBTB3 pathway; six in the Signalling by WNT pathway; and five in the Signalling by TGFB family members pathway (Figure 5B), according to the REACTOME classification. In Figure 5 we highlight using thick black borders some mRNAs found to be deregulated during the window of implantation (see Discussion).

## 3. Discussion

In mammalians, embryo implantation requires a receptive endometrium, a good-quality embryo, and a close dialogue between blastocyst and maternal tissues. The cross-communication between blastocyst and endometrial epithelial cells represents one of the most important and least-known aspects regulating the implantation process. It has been confirmed that both cell-free and extracellular vesicle (EV) cargo miRNAs represent key molecules able to mediate the dialogue between the embryo and maternal tissues [21]. In 2017, it was demonstrated that EVs present in human SCM are up-taken by human primary endometrial epithelial and stromal cells [22]; meanwhile, in 2023, it was observed that endometrial cells secrete EVs containing miRNAs related to implantation that are up-taken by human blastocysts [23].

MiRNAs, as regulators of gene expression, may control the activation or inhibition of biological pathways related to implantation in endometrial cells and their expression levels in the uterus in vivo or in SCM in vitro could define the suitable embryo for implantation [24]. Consequently, the possibility of using SCM miRNAs as non-invasive biomarkers of embryo quality and implantation success is the subject of a large number of papers [25,26,27]. Unfortunately, contradictory results are reported and, to date, the obtained data have not been translated into clinical practice [16]. This discrepancy in the literature data could depend on the difficulty in eliminating the different variables influencing implantation success. In fact, not only proper dialogue between blastocysts and maternal tissues, but also endometrium quality and embryo quality, contribute to and influence implantation and pregnancy rate [28]. Therefore, among the different variables, the miRNAs secreted by the embryo in SCM represent a limited fraction. Recently, our group demonstrated that six miRNAs are up-regulated in BF from implanted embryos with respect to non-implanted ones [20]. We previously suggested that a competent blastocyst, able to support the next steps of embryonic development, needs to secrete miRNAs in BF regulating the gene expression within the ICM or TE, particularly those involved in the pathways controlling pluripotency and differentiation [20]. In the same way, the miRNAs secreted outside the blastocyst could be involved in mediating the molecular dialogue between the embryo and maternal tissues [20].

In this paper, we aimed to highlight the different biological roles that miRNAs perform in BF and in SCM, independent of embryo implantation status, exploring the differences in miRNome in these two different compartments.

Among the ninety-six miRNAs analyzed, we found seventy-four miRNAs present in BF and/or in SCM. In more detail, fifty-eight miRNAs are shared by the two compartments, while ten and six of them are specifically expressed in BF and in SCM, respectively (Figure 1). Comparing the expression levels of the fifty-eight shared miRNAs, we found that twenty-two miRNAs were up-regulated in the comparison of BF vs. SCM, while two miRNAs (miR-19b and miR-320) were up-regulated in SCM (Table 1 and Figure 2). Interestingly, most of the miRNAs previously found to be up-regulated in BF from implanted embryos [20] were up-regulated in BF with respect to SCM, confirming their unique roles inside BF [20]. The miRNAs detected in SCM could be influenced by external contaminations derived from miRNAs present in blank culture medium (BM) [29,30]. To avoid this interference, we compared the expression levels of miRNAs specifically expressed in SCM or up-regulated in SCM with respect to BF with the miRNAs expressed in BM, and focused our attention on miRNAs significantly up-regulated in SCM with respect to BM (Figure 4). This approach allowed us to identify six miRNAs, which should be transcribed by the embryo and secreted in SCM (Figure 4A). This expression trend, validated by statistical analysis on four samples, was confirmed in the comparison of single BF and SCM coming from the same embryo (Figure 3). These results confirmed that the deregulation of the identified miRNAs does not depend on specific individual variations.

In order to validate the hypothesis that the miRNAs specifically expressed or up-regulated in SCM could regulate RNA transcripts (mRNAs) expressed in the endometrium during the implantation window, we retrieved the validated mRNA targets of SCM miRNAs, and we found that more than 50% of them (60/104) are expressed in the endometrium. Among them, twenty-one and sixteen mRNAs are involved in the Immune System and Developmental Biology pathways, respectively (Figure 5A). The immune system plays a crucial role in embryo implantation and the establishment of pregnancy. It is not simply a matter of suppressing the immune response, but rather a finely tuned process of immune regulation to both protect the embryo and ensure its successful implantation and development [31].

In our analysis, five miRNAs were able to regulate mRNAs included in signalling pathways involved in implantation, including ESR-mediated signalling; Signalling by Rho GTPases, Miro GTPases and RHOBTB3; Signalling by WNT; and Signalling by TGFB family members. The ESR-mediated signalling pathway plays a crucial role in embryo implantation, primarily through the action of oestrogen receptor alpha (ESR1) and its interaction with progesterone signalling. Oestrogen and progesterone, acting via their respective receptors (ESR1 and PGR), regulate the uterine environment to support blastocyst attachment and subsequent development [32]. Embryo implantation requires the dynamic coordination of cell migration, cytoskeletal remodelling and vesicle trafficking. Rho GTPases, are central regulators of actin cytoskeleton dynamics, cellular polarity, and migration, key processes in both uterine stromal cell decidualization and trophoblast invasion [33,34]. Their activity is regulated by Rho GTPase-activating proteins (RhoGAPs), such as ARHGAP1 (Rho GTPase-Activating Protein 1), which modulate the spatial and temporal activation of Rho proteins. While direct evidence linking ARHGAP1 to implantation remains limited, its functional role in regulating RhoA and Cdc42 suggests that it may indirectly influence implantation-related cytoskeletal rearrangements and cell motility [35]. Several studies have demonstrated that the Wnt signalling pathway is implicated in the regulation of uterine receptivity, pre-implantation embryo development, blastocyst activation for implantation, uterine development, and decidualization [36,37]. Finally, the role of TGF-β is important in many stages of pregnancy; it plays a role in embryo implantation by modulating decidualization, either enhancing or suppressing it, and by influencing key cellular processes, such as apoptosis and proliferation, that support successful implantation [38].

Several mRNAs targeted by the differentially expressed miRNAs and involved in the signalling pathways described above (Figure 5) were found to be down-regulated in the endometrium during the window of implantation [39]. This observation suggests that embryo-derived miRNAs, secreted into SCM, may contribute to this down-regulation. Among these mRNA targets, we identified key genes such as VEGFA (Vascular Endothelial Growth Factor A), ARHGAP1, CCND1 (Cyclin D1), DUT (Deoxyuridine Triphosphatase), and CREB1 (cAMP Responsive Element-Binding Protein 1) (Figure 5), each of which may play a pivotal role in implantation-related processes.

VEGFA plays a central role in the implantation process, by influencing both embryo development and endometrial receptivity. It is involved in angiogenesis and vascular permeability, which are essential for successful implantation [40,41]. ARHGAP1, a Rho GTPase-activating protein, contributes to cytoskeleton regulation and cell adhesion, both of which are fundamental to implantation. Rho GTPase-activating proteins (RhoGAPs) control Rho GTPase activity, essential for cytoskeletal dynamics and cell adhesion during this process [42].

CCND1 may be involved in the implantation process by regulating cell cycle progression. It regulates the transition from the G1 to the S phase of the cell cycle, and its expression is linked to both cell proliferation and differentiation [43].

DUT is required for maintaining nucleotide pool balance and genome integrity during cell proliferation, and is shown to be essential for implantation in murine models [44].

Finally, CREB1 plays a crucial role in the implantation process, particularly in the context of endometrial receptivity and decidualization. Alteration in CREB1 expression or activity can negatively impact endometrial receptivity, potentially leading to implantation failure [45,46].

In conclusion, although validation studies on a larger cohort of BF and SCM samples will be required to confirm these findings, our study identified six miRNAs, specifically detected in SCM, able to regulate endometrial mRNAs involved in implantation. We suggest that these miRNAs, performing a key role in the dialogue between the embryo and maternal tissues, could represent non-invasive biomarkers able to predict implantation success. We believe that their absence in SCM could represent a negative prognostic factor for implantation potential; however, their presence alone may not necessarily identify the most competent embryo. In fact, a competent embryo able to implant and lead to the birth of a healthy baby is the result of multifaceted processes including chromosome set up, and a proper quantity of RNA (mRNAs, non-coding RNAs), proteins, and different metabolites [47,48]. The modification of one of these aspects could result in lower embryonic competence and a decreased pregnancy rate. Moreover, it is important to analyze the different molecules (DNA, RNAs, and metabolites) in each embryo compartment.

For example, the presence of dysregulated miRNAs within BF does not mean that the same alteration will be detectable in the spent culture medium, and vice versa. This difference can be attributed to the distinct biological role played by miRNAs in these two compartments. In our previous paper [20] and in this one, we demonstrated that BF and SCM contain different miRNAs playing different and unique roles. Finally, the transcriptome will not necessarily be influenced by the presence of aneuploidies, and we can expect different RNA profiles based on the type of aneuploidy.

In summary, in our opinion there is no single “ideal” technique to identify the most competent embryo, but an integrated approach is needed to obtain informative results on the health of the embryo. Of course, in clinical practice, it will not be easy to obtain all this information; for this reason, the role of the gynaecologists or embryologists becomes important in directing the couple to carry out the most useful tests in a given clinical situation.

## 4. Materials and Methods

### 4.1. Sample Collection

We analyzed the expression profiles of ninety-six miRNAs in SCM and BF samples from the same embryo. Embryos were cultured individually in single drops (20 µL) of G-TL continuous culture medium by Vitrolife (Gothenburg, Sweden), specifically for the culture of human embryos from fertilization to the blastocyst stage, including embryo transfer, under a temperature of 37 °C, CO_2_ 6%, and oxygen 5% in a G210 InviCell incubator (Trumbull, Connecticut). All embryos were morphologically assessed on day three after fertilization (D3) and on day five after fertilization (D5). The embryo morphology assessment followed the Istanbul consensus workshop on embryo assessment. In total, eight (four SCM and four BF) samples from high-quality (4AA) embryos on day 5 post-fertilization were collected by Wunschbaby Institut Feichtinger (Vienna, Austria). For the collection of blastocoel fluid, an ICSI micropipette was used. The micropipette was inserted into the blastocoel cavity and the fluid was gently aspirated to induce blastocyst collapse prior to vitrification. The aspirated fluid was subsequently transferred into a 5 µL drop of ultrapure molecular biology-grade water and then into a 0.2 mL PCR tube. Samples were stored at −20 °C, without the addition of RNase inhibitors. Embryos were cultured in microdrops for 4–5 days before the collection of SCM. The collected SCM volume was 10 µL. Moreover, two samples of blank medium (BM), used as negative controls, were also collected. After collection, the samples were transferred to a 0.2 mL sterile PCR tube and stored at −80 °C for subsequent processing. The study was approved by the ethics committee Catania 1 (n. 178-2018-CA). The experiments were performed following the principles set out in the World Medical Association Declaration of Helsinki.

### 4.2. Design of Custom TaqMan Low-Density Array and miRNA Amplification

In our previously published paper, we identified eighty-nine miRNAs in BF by using TaqMan Low-Density Array (TLDA) technology (Panel A) (Applied Biosystem, by Thermo Fisher Scientific, Waltham, MA, USA), which allows the simultaneous analysis of up to 384 miRNAs per single sample [49]. Based on these findings, we used the eighty-nine expressed miRNAs to design a custom TLDA (Applied Biosystem, by Thermo Fisher Scientific, Waltham, MA, USA) specifically configured to analyze four samples in a single experiment. In Table S1 we report the custom card scheme.

RNA from the BF and SCM samples and from the BM was extracted by thermolysis, and we incubated the samples at 100 °C for 1 min to release nucleic acids, as previously described [49]. After this, 5 μL of each sample was directly reverse-transcribed and pre-amplified in final volumes of 15 μL and 50 μL, according to the manufacturer’s guideline for custom TLDAs. Quantitative RT-PCR reactions were performed on a QuantStudio 7 Flex Real-Time PCR System (Applied Biosystem, by Thermo Fisher Scientific, Waltham, MA, USA) with the following cycling conditions: 95 °C for 10 min, followed by 40 amplification cycles of 95 °C for 15 s and 60 °C for 1 min. Data analysis was conducted using QuantStudio Real-Time PCR software v1.3 (Applied Biosystem, by Thermo Fisher Scientific, Waltham, MA, USA) as previously reported [49].

### 4.3. Expression Data Analysis and Statistics

All miRNAs with Ct values between 15 and 35, detected in at least 50% of samples, were considered to be expressed and were included in the miRNA expression analysis. First, we compared miRNA expression levels between BF and SCM via the 2^−ΔΔCT^ method [50], using U6 small nuclear RNA as the endogenous control for data normalization [20]. Then, the miRNAs specifically expressed or up-regulated in SCM were compared with respect to BM miRNAs, in order to identify potential medium-derived contamination.

The normal distribution of values was assessed by a Shapiro–Wilk test. The unpaired *t*-test was applied to statistically evaluate miRNA expression differences between the compared groups. Statistical significance was assessed by setting the *p*-value cut-off at ≤0.05, without performing any *p*-value adjustment tests [51].

### 4.4. Network miRNA–mRNA Interaction and Enrichment Pathway Analysis

In order to evaluate the biological role of the miRNAs specifically expressed or significantly overexpressed in SCM, their validated target genes were retrieved from miRTarBase (https://awi.cuhk.edu.cn/~miRTarBase/miRTarBase_2025/php/index.php, accessed on 9 June 2025).

The identified targets were subjected to functional enrichment analysis using the Database for Annotation, Visualization and Integrated Discovery (DAVID) tool (https://davidbioinformatics.nih.gov/, accessed on 11 June 2025). The analysis was restricted to mRNAs expressed in the endometrium. From this subset, genes involved in biologically relevant REACTOME pathways were selected. The final list of pathway-associated, endometrium-expressed target genes was used to construct an interaction network, providing insight into the potential regulatory mechanisms mediated by the selected miRNAs.

## Figures and Tables

**Table 1:** Differentially expressed miRNAs in BF vs. SCM. Ln (FC), standard deviation (±std. dev), and *p*-value are reported.

miRNA ID	Ln (FC)	±Std. Dev	*p*-Value
miR-106a	5.13	0.76	0.001
miR-141	4.70	1.25	0.005
miR-150	4.18	1.10	0.001
miR-17	3.53	1.28	0.02
miR-192	4.04	1.01	0.001
miR-19b	−4.01	1.24	0.01
miR-202	5.73	1.44	0.0005
miR-203	4.38	1.29	0.001
miR-20a	5.28	1.30	0.0004
miR-212	3.26	2.33	0.04
miR-26a	3.73	2.19	0.02
miR-29c	6.05	1.33	0.0005
miR-302a	7.19	1.57	0.0001
miR-302b	7.05	1.33	0.01
miR-302c	2.55	1.10	0.01
miR-30c	3.41	0.74	0.0001
miR-31	4.90	1.05	0.004
miR-320	−2.00	0.96	0.04
miR-345	7.08	1.29	0.0013
miR-367	7.00	1.16	0.0009
miR-373	6.74	1.53	0.0002
miR-512-3p	4.02	1.07	0.01
miR-515-3p	6.76	1.25	0.01
miR-597	5.84	1.50	0.0004

## Data Availability

The data that support the findings of this study are available from the corresponding author (angela.caponnetto@unict.it) upon reasonable request.

## Supplementary Materials

The following supporting information can be downloaded at: https://www.mdpi.com/article/10.3390/ijms26188879/s1.
