# Supplementary material for: MicroRNAs Secreted by the Embryo in Spent Culture Medium Can Regulate mRNAs Involved in Endometrial Receptivity, Embryo Attachment, and Invasion
Source: Int J Mol Sci. 2025 Sep 12;26(18):8879. doi: 10.3390/ijms26188879 (PMC12469383; doi:10.3390/ijms26188879)
Supplement: Supplementary file 1 [file ijms-26-08879-s001.zip › ijms-3835652-supplementary.pdf]

Table S1. Custom Card configuration

Detector	Assay	ID	Type
**A1**	ath-miR159a-000338	338	Negative Control
**A2**	hsa-miR-106a-002169	2169	Endogenous
**A3**	hsa-miR-106b-000442	442	Endogenous
**A4**	hsa-miR-125a-5p-002198	2198	Endogenous
**A5**	hsa-miR-126-002228	2228	Endogenous
**A6**	hsa-miR-130a-000454	454	Endogenous
**A7**	hsa-miR-132-000457	457	Endogenous
**A8**	hsa-miR-136-000592	592	Endogenous
**A9**	hsa-miR-138-002284	2284	Endogenous
**A10**	hsa-miR-141-000463	463	Endogenous
**A11**	U6 snRNA-001973	1973	Housekeeping
**A12**	U6 snRNA-001973	1973	Housekeeping
**A13**	hsa-miR-142-3p-000464	464	Endogenous
**A14**	hsa-miR-146a-000468	468	Endogenous
**A15**	hsa-miR-146b-001097	1097	Endogenous
**A16**	hsa-miR-150-000473	473	Endogenous
**A17**	hsa-miR-155-002623	2623	Endogenous
**A18**	hsa-miR-15b-000390	390	Endogenous
**A19**	hsa-miR-16-000391	391	Endogenous
**A20**	hsa-miR-17-002308	2308	Endogenous
**A21**	hsa-miR-184-000485	485	Endogenous
**A22**	hsa-miR-191-002299	2299	Endogenous
**A23**	hsa-miR-192-000491	491	Endogenous
**A24**	hsa-miR-193b-002367	2367	Endogenous
**B1**	hsa-miR-194-000493	493	Endogenous
**B2**	hsa-miR-195-000494	494	Endogenous
**B3**	hsa-miR-197-000497	497	Endogenous
**B4**	hsa-miR-19a-000395	395	Endogenous
**B5**	hsa-miR-19b-000396	396	Endogenous
**B6**	hsa-miR-200c-002300	2300	Endogenous
**B7**	hsa-miR-202-002363	2363	Endogenous
**B8**	hsa-miR-203-000507	507	Endogenous
**B9**	hsa-miR-204-000508	508	Endogenous
**B10**	hsa-miR-205-000509	509	Endogenous
**B11**	hsa-miR-20a-000580	580	Endogenous
**B12**	hsa-miR-20b-001014	1014	Endogenous
**B13**	hsa-miR-212-000515	515	Endogenous
**B14**	hsa-miR-222-002276	2276	Endogenous
**B15**	hsa-miR-223-002295	2295	Endogenous
**B16**	hsa-miR-24-000402	402	Endogenous
**B17**	hsa-miR-26a-000405	405	Endogenous
**B18**	hsa-miR-28-3p-002446	2446	Endogenous
**B19**	hsa-miR-29a-002112	2112	Endogenous
**B21**	hsa-miR-301-000528	528	Endogenous
**B20**	hsa-miR-29c-000587	587	Endogenous

**B22**	hsa-miR-302a-000529	529	Endogenous
**B23**	hsa-miR-302b-000531	531	Endogenous
**B24**	hsa-miR-302c-000533	533	Endogenous
**C1**	hsa-miR-30b-000602	602	Endogenous
**C2**	hsa-miR-30c-000419	419	Endogenous
**C3**	hsa-miR-31-002279	2279	Endogenous
**C4**	hsa-miR-320-002277	2277	Endogenous
**C5**	hsa-miR-342-3p-002260	2260	Endogenous
**C6**	hsa-miR-345-002186	2186	Endogenous
**C7**	hsa-miR-34a-000426	426	Endogenous
**C8**	hsa-miR-362-001273	1273	Endogenous
**C9**	hsa-miR-367-000555	555	Endogenous
**C10**	hsa-miR-371-3p-002124	2124	Endogenous
**C11**	hsa-miR-372-000560	560	Endogenous
**C12**	hsa-miR-373-000561	561	Endogenous
**C13**	hsa-miR-374-000563	563	Endogenous
**C14**	hsa-miR-381-000571	571	Endogenous
**C15**	hsa-miR-410-001274	1274	Endogenous
**C16**	hsa-miR-454-002323	2323	Endogenous
**C17**	hsa-miR-484-001821	1821	Endogenous
**C18**	hsa-miR-512-3p-001823	1823	Endogenous
**C19**	hsa-miR-515-3p-002369	2369	Endogenous
**C20**	hsa-miR-515-5p-001112	1112	Endogenous
**C21**	hsa-miR-517a-002402	2402	Endogenous
**C22**	hsa-miR-517b-001152	1152	Endogenous
**C23**	hsa-miR-517c-001153	1153	Endogenous
**C24**	hsa-miR-518a-3p-002397	2397	Endogenous
**D1**	hsa-miR-518b-001156	1156	Endogenous
**D2**	hsa-miR-518d-001159	1159	Endogenous
**D3**	hsa-miR-518e-002395	2395	Endogenous
**D4**	hsa-miR-518f-002388	2388	Endogenous
**D5**	hsa-miR-519a-002415	2415	Endogenous
**D6**	hsa-miR-519d-002403	2403	Endogenous
**D7**	hsa-miR-520a#-001168	1168	Endogenous
**D8**	hsa-miR-520b-001116	1116	Endogenous
**D9**	hsa-miR-520d-5p-002393	2393	Endogenous
**D10**	hsa-miR-520g-001121	1121	Endogenous
**D11**	hsa-miR-522-002413	2413	Endogenous
**D12**	hsa-miR-525-3p-002385	2385	Endogenous
**D13**	hsa-miR-526b-002382	2382	Endogenous
**D14**	hsa-miR-532-001518	1518	Endogenous
**D15**	hsa-miR-548a-001538	1538	Endogenous
**D16**	hsa-miR-548c-001590	1590	Endogenous
**D17**	hsa-miR-590-5p-001984	1984	Endogenous
**D18**	hsa-miR-597-001551	1551	Endogenous
**D19**	hsa-miR-886-3p-002194	2194	Endogenous
**D20**	hsa-miR-886-5p-002193	2193	Endogenous

**D21**	hsa-miR-9-000583	583	Endogenous
**D22**	hsa-miR-92a-000431	431	Endogenous
**D23**	RNU44-001094	1094	Positive Control
**D24**	RNU48-001006	1006	Positive Control
